# Screening for colorectal cancer: random comparison of guaiac and immunochemical faecal occult blood testing at different cut-off levels

**DOI:** 10.1038/sj.bjc.6604961

**Published:** 2009-03-31

**Authors:** L Hol, J A Wilschut, M van Ballegooijen, A J van Vuuren, H van der Valk, J C I Y Reijerink, A C M van der Togt, E J Kuipers, J D F Habbema, M E van Leerdam

**Affiliations:** 1 Department of Gastroenterology and Hepatology, Erasmus MC, University Medical Centre, Rotterdam, The Netherlands; 2 Department of Public Health, Erasmus MC, University Medical Centre, Rotterdam, The Netherlands; 3 Association of Laboratory Pathology and Cytology (Pathan), Rotterdam, The Netherlands; 4 Association of Nation-wide Screening South-west Netherlands, Vlaardingen, The Netherlands; 5 Comprehensive Cancer Centre, Rotterdam, The Netherlands; 6 Internal Medicine, Erasmus MC, University Medical Centre, Rotterdam, The Netherlands

**Keywords:** colorectal cancer screening, immunochemical faecal occult blood test, guaiac-based faecal occult blood test, randomised trial, population based, cut-off

## Abstract

Immunochemical faecal occult blood testing (FIT) provides quantitative test results, which allows optimisation of the cut-off value for follow-up colonoscopy. We conducted a randomised population-based trial to determine test characteristics of FIT (OC-Sensor micro, Eiken, Japan) screening at different cut-off levels and compare these with guaiac-based faecal occult blood test (gFOBT) screening in an average risk population. A representative sample of the Dutch population (*n*=10 011), aged 50–74 years, was 1 : 1 randomised before invitation to gFOBT and FIT screening. Colonoscopy was offered to screenees with a positive gFOBT or FIT (cut-off 50 ng haemoglobin/ml). When varying the cut-off level between 50 and 200 ng ml^−1^, the positivity rate of FIT ranged between 8.1% (95% CI: 7.2–9.1%) and 3.5% (95% CI: 2.9–4.2%), the detection rate of advanced neoplasia ranged between 3.2% (95% CI: 2.6–3.9%) and 2.1% (95% CI: 1.6–2.6%), and the specificity ranged between 95.5% (95% CI: 94.5–96.3%) and 98.8% (95% CI: 98.4–99.0%). At a cut-off value of 75 ng ml^−1^, the detection rate was two times higher than with gFOBT screening (gFOBT: 1.2%; FIT: 2.5%; *P*<0.001), whereas the number needed to scope (NNscope) to find one screenee with advanced neoplasia was similar (2.2 *vs* 1.9; *P*=0.69). Immunochemical faecal occult blood testing is considerably more effective than gFOBT screening within the range of tested cut-off values. From our experience, a cut-off value of 75 ng ml^−1^ provided an adequate positivity rate and an acceptable trade-off between detection rate and NNscope.

Colorectal cancer (CRC) is a major health problem in the Western world. Screening can reduce CRC mortality due to detection of early carcinomas and removal of pre-malignant lesions (Winawer *et al*, 1993; Ries *et al*, 2007). The American Gastroenterology Association (Winawer *et al*, 2003), the US Multi-Society Task Force (Levin *et al*, 2008), Asia Pacific Working Group on Colorectal Cancer screening (Sung *et al*, 2008) and the European council (Council Recommendation on Cancer Screening, 2003) recommend CRC screening for average risk individuals over 50 years of age. Several countries have a nation-wide screening programme mainly based on guaiac-based faecal occult blood test (gFOBT), as this is the only available test with a proven mortality reduction (Mandel *et al*, 1993; Hardcastle *et al*, 1996; Kronborg *et al*, 1996), but consider changing to an immunochemical FOBT (FIT) programme based on accumulating evidence that FIT is superior to gFOBT screening, including a higher attendance (Cole *et al*, 2003; van Rossum *et al*, 2008; Hol *et al*, 2008) and detection rate (Smith *et al*, 2006; Guittet *et al*, 2007; van Rossum *et al*, 2008), as well as a higher sensitivity without a significant drop in specificity (Allison *et al*, 1996, 2007; Wong *et al*, 2003; Morikawa *et al*, 2005; Guittet *et al*, 2007; Levi *et al*, 2007). Furthermore, FIT specifically binds human haemoglobin (Hb), which makes drugs and diet restrictions superfluous.

Immunochemical faecal occult blood testing samples can be analysed automatically, which has important advantages for reproducibility, quality control, capacity, and thus personnel need and costs (Young *et al*, 2002; Levi *et al*, 2007). Another advantage of FIT is the quantitative test results, which allows determining an optimal cut-off value for a nation-wide screening programme (Castiglione *et al*, 2002; Wong *et al*, 2003; Guittet *et al*, 2007; Levi *et al*, 2007; Fraser *et al*, 2008). The cut-off value for a positive test can be based on a positivity rate that meets the available colonoscopy resources. At the same time, the number of colonoscopies is an important determinant of the neoplasia detection rate, and thus of the potential preventive effect of a CRC screening programme.

Data on positivity rate and test performance at different cut-off levels of FIT screening in an average risk population are highly needed to determine the optimal cut-off value for FIT screening. We, therefore, conducted a randomised trial to compare the positivity rate, detection rate and specificity of FIT (OC-Sensor micro; Eiken Chemical Co., Tokyo, Japan) screening at different cut-off levels with gFOBT (Hemoccult II; Beckman Coulter Inc., Fullerton, CA, USA) screening in an average risk screening-naive population.

## Materials and methods

### Study population

**Figure 1:**
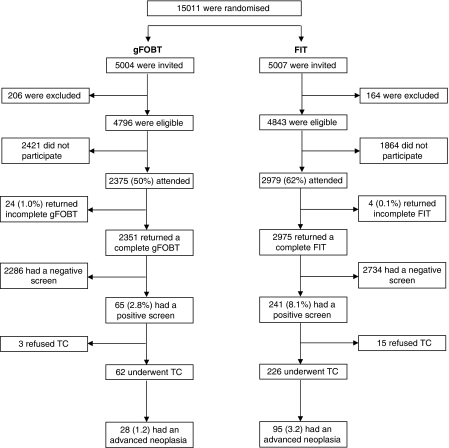
Trial profile. gFOBT: guaiac-based faecal occult blood test; FIT: immunochemical faecal occult blood test; TC: total colonoscopy.

The study was performed in the Rijnmond region in the southwest of the Netherlands. This region includes Rotterdam and surrounding villages and harbours 338 000 inhabitants in the target population. The region thus combines both rural and urban settings. Ten thousand and eleven individuals, aged 50–74 years, were randomly selected from the municipal registries. The selected individuals were 1 : 1 randomised per household after stratifying for age, sex and social economic status into group A (gFOBT) or B (FIT) using a computer-generated allocation algorithm (Tenalea, Amsterdam, The Netherlands) (Figure 1). Randomisation occurred before invitation. Informed consent was asked after randomisation. Individuals with a history of inflammatory bowel disease or CRC, a colonoscopy, sigmoidoscopy or barium contrast enema in the last 3 years, major health problems or inability to sign informed consent were excluded. Recruitment took place between November 2006 and November 2007.

### Interventions

The randomly selected 10 011 individuals were sent a pre-invitation letter containing information on CRC screening. Two weeks later, an invitation letter was sent with information on possible advantages and risks of screening. This was accompanied by an informed consent form that had to be signed and returned. A test set was sent along with the invitation. A reminder was sent 6 weeks afterwards to all non-respondents. Information about the study was further given by direct visits of research physicians to all general practitioners (GPs) in the region, as well as through a dedicated website (www.dikkedarmkankerpreventie.nl), mailings and information sites of the municipality offices, regional newspapers, and national and regional broadcasting.

### Group A: gFOBT

All individuals randomised to gFOBT received three guaiac imprinted test cards (Hemoccult II) to be used with three consecutive bowel movements without dietary restrictions or medication limitations. Participants returned the test kit by mail to the Gastroenterology and Hepatology laboratory of the Erasmus University Medical Centre. Tests were analysed without re-hydration. A test was considered positive if at least one of six panels was positive. A digital picture of test cards was taken and stored in a database. As a quality control, 241 (10%) photographs were re-evaluated by a second technician blinded for the initial test results. A third technician reviewed the photographs in case of inter-observer variation.

### Group B: immunochemical FOBT

Subjects randomised to FIT screening received one FIT kit (OC-Sensor micro) to collect a single faecal sample of one bowel movement without dietary restrictions or medication limitations. Participants returned the test kit by mail to the same laboratory that analysed the gFOBT for quantitative analysis using the automatic OC-Sensor micro instrument. Participants were referred to colonoscopy at Hb levels above 50 ng ml^−1^.

### Follow-up

In case of a negative gFOBT or FIT, both the GP and the participant were informed by mail within 3 weeks. No further follow-up was necessary. In case of a positive gFOBT or FIT (faecal Hb level ⩾50 ng ml^−1^), the GP was informed both by telephone and mail within 2 weeks. The GP informed the participant about the test result and referred the participant for colonoscopy. A colonoscopy was scheduled within 2 weeks after the screening test results had become available.

### Colonoscopy

All colonoscopies were performed in eight hospitals and performed by experienced endoscopists (individual experience >200 colonoscopies per annum). The reach of the endoscope in cm and the location, as well as the adequacy of bowel preparation, were recorded. During colonoscopy, characteristics, including size, pedunculated or sessile aspect and location of all polyps, were noted and recorded. Location was defined as rectum, sigmoid, descending, transverse, ascending colon or caecum, and was measured in cm from the anal verge with the endoscope in the straightened position. Size of each polyp was estimated using an open biopsy forceps with a span of 7 mm. An experienced gastrointestinal pathologist evaluated all removed polyps. In accordance with the international classification, CRC was defined as the invasion of malignant cells beyond the muscularis mucosa. Patients with intramucosal carcinoma or carcinoma *in situ* were classified as having high-grade dysplasia.

### Ethical approval

The study was approved by the Dutch Ministry of Health (2006/02WBO). The approval included the pre-randomisation design. The study letters and information brochures were approved by the Institutional Review Board of the Erasmus MC (MEC-2005-264).

### Statistical analysis

Differences in proportions between screening strategies were calculated using a *χ*^2^ test. Differences in means between screening strategies were calculated using a Student's *t*-test. All *P*-values were two-sided and considered significant if <0.05. Uni- and multivariate logistic regression analyses were used to determine the influence of sex and age on positivity rate, number needed to scope (NNscope), detection rate and number needed to screen (NNscreen). The positivity rate was defined as the proportion of participants having a positive gFOBT or FIT test. For FIT, the positivity rate was separately calculated for cut-off levels of 50, 75, 100, 125, 150, 175 and 200 ng ml^−1^, respectively. The detection rate was defined as the proportion of participants having advanced neoplasia. This was calculated as the number of screenees with an advanced neoplasia divided by all screenees with a complete screening test. Advanced neoplasia included CRC and advanced adenoma. Advanced adenoma was defined as adenoma ⩾10 mm or with a histology showing either a ⩾25% villous component or high-grade dysplasia. We compared faecal Hb measurements between screenees with a normal colonoscopy and screenees with non-neoplastic polyps, non-advanced adenomas and advanced adenomas and CRC as the most advanced lesion by the Kruskal–Wallis non-parametric analysis of variance and the Mann–Whitney test, as the data were not normally distributed. Participation, positivity and detection rate, positive predictive value (PPV) and specificity were calculated and described as percentages with 95% confidence intervals (95% CI). The specificity for advanced neoplasia and CRC was calculated under the rare disease assumption as the ratio of the number of all negative screenees and the total number of screenees subtracted by the number of true positives (Brecht and Robra, 1987). Number needed to scope describes the number of colonoscopies to find one screenee with an advanced neoplasia or CRC. Number needed to screen was calculated as the number of complete screening tests needed to find one advanced neoplasia or CRC. Differences in PPV between sexes or age groups in the FIT arm were described for a cut-off of 100 ng ml^−1^, as this cut-off value is most commonly used (Castiglione *et al*, 2002; Vilkin *et al*, 2005; van Rossum *et al*, 2008).

## Results

In total, 10 011 subjects were randomised before invitation to one of the two FOBTs. Three hundred and seventy (3.7%) subjects were excluded from analyses (332 subjects met one of the exclusion criteria, 26 had moved away and 12 had died). A total of 2375 out of 4796 (50%; 95% CI: 48–51%) participants attended gFOBT screening. The gFOBT was analysable in 2351 cases (99%). In all, 2979 out of 4843 (62%; 95% CI: 60–63%) subjects attended FIT screening and the test was complete in 2975 subjects (99.9%) (Figure 1). The distribution of age (mean±s.d. gFOBT 61±7 years; FIT 61±7 years old) and sex (male gFOBT 46%; FIT 48%) of the analysable subjects did not differ between the two screening arms.

### Proportion of positive tests

**Figure 2:**
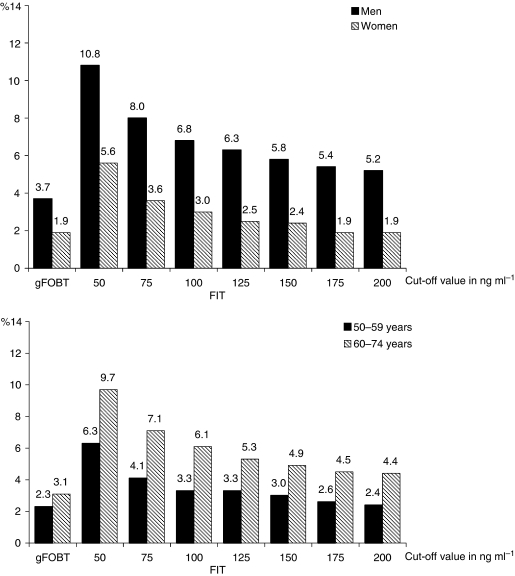
Positivity rate of gFOBT and FIT at different cut-offs in men and women aged 50–59 and 60–74 years.

In total, 65 screenees had a positive gFOBT (2.8%; 95% CI: 2.2–3.6%). Immunochemical faecal occult blood testing was positive in 241 screenees (8.1%; 95% CI: 7.2–9.1%) at a cut-off of 50 ng ml^−1^ and in 103 screenees (3.5%; 95% CI: 2.9–4.2%) at a cut-off of 200 ng ml^−1^ (Table 1). A significant decrease in the proportion of positive tests was seen between cut-off values of 50 and 75 ng ml^−1^ (8.1 *vs* 5.7%), followed by a more gradual decrease between cut-off values of 75 and 200 ng ml^−1^ (Table 1). Male screenees were more likely to have a positive gFOBT than female screenees (3.7 *vs* 1.9%; OR: 1.4; CI: 1.1–1.8) or FIT (FIT^100^: 6.8 *vs* 3.0%; OR: 2.3; 95% CI: 1.6–3.3). The proportion of positive gFOBTs was slightly higher in screenees aged 60–74 years than in screenees aged 50–59 years, but this difference was not significant (3.1 *vs* 2.3%; OR: 1.3; 95% CI: 0.8–2.2). In the FIT arm, the proportion of positive tests was significantly higher in screenees aged 60–74 years than in screenees aged 50–59 years (FIT^100^: 6.1 *vs* 3.3%; OR: 1.8; 95% CI: 1.3–2.6) (Figure 2).

### Colonoscopy findings per test and cut-off value

Sixty-two (95.4%) of the 65 gFOBT-positive screenees and 226 (93.8%) of the 241 screenees with an FIT result ⩾50 ng ml^−1^ underwent a colonoscopy. A double-contrast barium enema was performed in three subjects with an incomplete colonoscopy. Two colonoscopies were incomplete due to an obstructing tumour. The colonoscopy findings are in Table 2 and are related to the amount of Hb in the faeces. A significantly higher proportion of screenees with faecal Hb levels of 150–200 (47%) and ⩾200 (61%) had advanced neoplasia than screenees with faecal Hb levels of 50–150 ng (25%) (*P*=0.009 and *P*<0.001, respectively), whereas the proportions were similar among screenees with values of 50–100 ng ml^−1^ and 100–150 ng ml^−1^ (25 *vs* 18%; *P*=0.60).

### Haemoglobin levels per finding

The median faecal Hb level of positive screenees with a normal colonoscopy was 50 ng ml^−1^. Median Hb measurement in screenees with, as the most advanced finding, a non-neoplastic polyp was 94 ng ml^−1^, with a non-advanced adenoma was 112 ng ml^−1^, with an advanced adenoma was 373 ng ml^−1^ and with a CRC was 404 ng ml^−1^. Faecal Hb levels of screenees with a normal colonoscopy did not significantly differ from those of screenees with non-neoplastic (*P*=0.88) or non-advanced adenoma (*P*=0.89), whereas the faecal Hb level of screenees with an advanced adenoma or CRC was significantly higher than that of screenees with a normal colonoscopy (both *P*<0.001). The difference in feacal Hb level between those with advanced adenoma and those with CRC was not significant (*P*=0.53).

### Test characteristics

The PPV of gFOBT for advanced neoplasia and for CRC was 45% (95% CI: 33–58%) and 10% (95% CI: 4–20%), respectively. Immunochemical faecal occult blood testing showed a more favourable PPV for detecting advanced neoplasia at higher cut-off values (Table 1), but this difference was only significant at cut-off values ⩾175 ng ml^−1^ (gFOBT 45% *vs* FIT^175^ 63%; *P*=0.029 and FIT^200^ 62%; *P*=0.035). The PPV for CRC was similar for gFOBT and FIT at all cut-off levels, although the PPV of FIT steadily increased with increasing cut-off value (Table 1).

**Figure 3:**
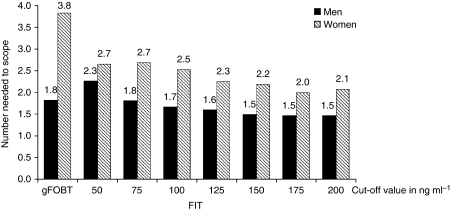
Numbers needed to scope to find one screenee with an advanced neoplasia in men and women at different cut-off values.

The NNscope to detect one screenee with an advanced neoplasia or CRC was 2.2 and 10.3, respectively, for gFOBT. The corresponding numbers with FIT screening were 2.4 and 14.1 at 50 ng ml^−1^ and 1.6 and 8.2 at 200 ng ml^−1^ cut-off values (Table 1) for advanced neoplasia and CRC, respectively. Men showed a lower NNscope for advanced neoplasia than women (gFOBT men: 1.8; women: 3.8; *P*=0.04; FIT^100^: 1.7; women: 2.5; *P*=0.03) (Figure 3). No differences in NNscope for advanced neoplasia or CRC were seen between different age groups (gFOBT, *P*=0.33; FIT^100^, *P*=0.81).

The estimated specificity for not having advanced neoplasia and CRC was significantly lower for FIT at cut-off values ⩽100 ng ml^−1^ than that for gFOBT (Table 1). Above a cut-off value of 100 ng ml^−1^, the estimated specificity was similar to that of gFOBT.

### Detection rate

In the range of tested cut-off levels, FIT detected more advanced neoplasia than gFOBT (gFOBT: 1.2%; 95% CI: 0.8–1.7%; FIT^50^: 3.2%; 95% CI: 2.6–3.9%; FIT^200^: 2.1%; 95% CI: 1.6–2.6%), whereas similar detection rates for CRC were found for gFOBT and FIT screening.

Male sex was associated with a higher detection rate of advanced neoplasia in both screening arms (gFOBT: OR 4.2; 95% CI: 1.7–10.4; FIT^100^: OR 3.5; 95% CI: 2.0–6.1). Screenees aged 60–74 years showed a higher detection rate of advanced neoplasia than screenees aged 50–59 years in the FIT arm (FIT^100^: OR 1.9; 95% CI: 1.2–3.2), whereas no significant difference between both age groups was found in the gFOBT arm (OR 1.5; 95% CI: 0.7–3.3).

The NNscreen to find at least one advanced neoplasia was favourable at all cut-off levels for FIT compared with the gFOBT arm (Table 1). Male screenees showed significantly lower numbers needed to screen to detect one advanced neoplasia than female screenees (gFOBT: men: 57 *vs* women: 181; *P*=0.002; FIT 100^100^: men: 26 *vs* women: 91; *P*<0.001).

## Discussion

We compared FIT screening at different cut-off levels with conventional gFOBT screening in an average risk screening-naive population. Our results show that FIT within the complete range of tested cut-off values (50–200 ng ml^−1^) outperforms gFOBT screening as it is associated with both higher attendance as well as higher detection rates of advanced neoplasia, even though the PPV for detecting advanced neoplasia did not differ significantly between both tests. The outperformance of FIT over gFOBT on both attendance and yield is very relevant for the potential impact of faecal occult blood-based screening on mortality due to CRC.

Furthermore, FIT testing provides quantitative results, which allows the determination of an optimal cut-off value for a nation-wide screening programme based on colonoscopy capacity and the intended detection rate in the screened population. A low cut-off value (50 ng ml^−1^) provided not only a high detection rate of advanced neoplasia, but also more false-positive test results and thus a higher number of unnecessary colonoscopies. False-positive results are associated with anxiety (Taylor *et al*, 2004) and increased costs (Castiglione *et al*, 1997). Increasing the cut-off value resulted in a decrease in detection rate but a more favourable PPV. The key question is at which cut-off value the magnitude of benefits (possible the early detection of CRC or the removal of adenomas) is sufficient to outweigh the harms (burden, complications, demand on colonoscopy capacity and costs of screening). The cut-off at which this trade-off becomes acceptable must be determined in a full cost-effectiveness analysis. However, the ratio between detection rate and NNscope to find one screenee with an advanced neoplasia is a good indicator for this trade-off, as it reflects both benefit (detecting an advanced neoplasia) and harm (the need to undergo colonoscopy). We found that the NNscope was higher with FIT than with gFOBT screening when using an FIT cut-off of 50 ng ml^−1^, but this changed in favour of FIT when increasing the cut-off to 75 ng ml^−1^ (Table 1). At a cut-off value of 75 ng ml^−1^ , the detection rate with FIT was two-fold higher than that with gFOBT. At the same time, increasing the FIT cut-off from 50 to 75 ng ml^−1^ had a considerably stronger limiting effect on the proportion of FIT positives (falling from 8.1 to 5.7%) than any other similar further increase of the FIT cut-off (Table 1). Further increasing the cut-off level from 75 to 100 ng ml^−1^ would result in a larger decline in detection rate (8.8%) than in NNscope (7.3%) and therefore a less favourable trade-off (Table 1). For these reasons, we conclude that FIT provided the most optimal trade-off when using a cut-off value of 75 ng ml^−1^. This conclusion is in agreement with observations from a colonoscopy study determining the one-time sensitivity and specificity of the same OC-Micro Latex FIT test in a population of individuals at higher risk for CRC (Levi *et al*, 2007). The latter study and our results come to a lower cut-off than the recommended cut-off value of 100 ng ml^−1^ by the manufacturer (Eiken Chemical Co.) and by an earlier study examining the performance of the OC-Sensor at different cut-off levels (Castiglione *et al*, 2000).

Our findings on positivity rate, PPV and the detection rate of CRC at a cut-off value of 100 ng ml^−1^ are in agreement with those of other studies using the OC-Sensor with this specific cut-off (Castiglione *et al*, 2000, 2002; Grazzini *et al*, 2004; Dancourt *et al*, 2008; van Rossum *et al*, 2008). Both our study and a similarly designed study by van Rossum *et al* (2008), however, found a significantly higher PPV and detection rate for advanced neoplasia (PPV: 52–53%; DR: 2.4–2.5%) than other studies (PPV: 20–39%; DR: 0.8–1.2%) (Castiglione *et al*, 2000, 2002; Grazzini *et al*, 2004; Dancourt *et al*, 2008), even though these studies all focused on the same age group and applied the same test and definition of advanced neoplasia. A possible explanation is that both Dutch studies were carried out in a screening-naive population, whereas other studies from Italy and France (Castiglione *et al*, 2000, 2002; Grazzini *et al*, 2004; Dancourt *et al*, 2008) were performed in parallel to a nation-wide programme and therefore were more likely to have included subjects screened earlier subjects with a lower risk on advanced neoplasia.

The positivity rate is the main driver for the number of colonoscopies among attendants. In countries with a gFOBT screening programme, changing to FIT screening with a 50 ng ml^−1^ cut-off value would require a considerable (gFOBT: 2.8% *vs* FIT^50^ 8.1% positivity rate) increase in colonoscopy capacity for screening. This effect is augmented by a higher attendance rate to FIT than to gFOBT screening (van Rossum *et al*, 2008; Hol *et al*, 2008). Thus, FIT screening enables a more efficient screening with increased participation (Cole *et al*, 2003; van Rossum *et al*, 2008; Hol *et al*, 2008) and improved test performances (Allison *et al*, 1996, 2007; Greenberg *et al*, 2000; Zappa *et al*, 2001; Smith *et al*, 2006; Guittet *et al*, 2007; van Rossum *et al*, 2008), potentially allowing a decrease in screening intensity by lengthening the screening interval.

The detection rate of advanced neoplasia was significantly higher in men than in women in both screening arms. Likewise, the NNscreen to detect an advanced neoplasia was lower in men than in women. Similar differences in detection rates for advanced neoplasia between both sexes were found in two colonoscopy screening studies (Lieberman *et al*, 2000; Schoenfeld *et al*, 2005; Regula *et al*, 2006). Furthermore, the CRC incidence rates are on an average 1.5 times higher in men than in women aged 50–75 years (Ries *et al*, 2007; Jemal *et al*, 2008). Thus, the higher pre-test probabilities for advanced neoplasia in men explain this difference. Several studies have, therefore, suggested to develop sex-specific recommendations for CRC screening (Lieberman, 2005; Brenner *et al*, 2007). A differentiated approach taking sex and potentially age into account would be relatively easy with FIT screening. One could argue to use different cut-off values for men and women to achieve a similar NNscope, which would result in a considerable higher cut-off value for women than for men (Figure 3).

This study was not designed to estimate the sensitivity and specificity of FOBT, as negative screenees did not undergo a colonoscopy (golden standard). The aim of this study was to compare test characteristics of gFOBT and FIT at different cut-off values. The detection rate and false-positive test results could be used as an indication for test sensitivity and specificity, respectively, as both tests were performed in a similar population. Specificity for advanced neoplasia of gFOBT and FIT was estimated under the rare disease assumption based on the number of false-positive screenees. The specificity can be overestimated if the number of false negatives increases, which is seen in diseases with a high prevalence and more sensitive tests (Brecht and Robra, 1987). Therefore, the specificity of advanced adenoma could be slightly overestimated in both screening arms due to a higher prevalence. Another limitation of the design of this study is that the mean Hb levels per lesion (non-neoplastic polyp, non-advanced adenoma, advanced adenoma or CRC) only pertain to screenees who had a positive test (faecal Hb level ⩾50 ng ml^−1^) and subsequently underwent a follow-up colonoscopy. These results can, therefore, not be generalised to all screenees. However, this observation could be used for prioritising of colonoscopies in subjects with a positive test, a topic that can be very relevant in areas and at time periods of shortage of endoscopic capacity, even when all subjects with a test result above a chosen cut-off should undergo endoscopy within a limited time span. Furthermore, this study describes the first screening round in our population. Data on PPV and detection rate of successive screening rounds are needed to provide an insight into the long-term effectiveness of a population-based screening programme.

In conclusion, this randomised population-based trial provides important data on the test characteristics of FIT screening at different cut-off values. Immunochemical faecal occult blood testing screening is considerably more effective than gFOBT within the complete range of tested cut-off values. From our experience, a cut-off value of 75 ng ml^−1^ provided an adequate positivity rate and an acceptable trade-off between detection rate and NNscope to find a screenee with an advanced neoplasia. Increasing the cut-off value can be considered in case of insufficient colonoscopy capacity, at the cost of a gradual decrease in detection rate. The optimal cut-off value within a specific population can be based on a local screening programme, taking major determinants into account, including the incidence of neoplasia, the intended screening interval, colonoscopy capacity and cost efficacy. With this in mind, the use of variable cut-offs for different sub-groups is a further option for individualised CRC screening.

## Figures and Tables

**Table 1:** Test characteristics of gFOBT and FIT at different cut-off levels

	**Cut-off**	**Positivity rate**		**PPV**		**NNscope**		**Specificity**		**Detection rate**				**NNscreen**	
	**(ng ml^−1^)**	***n***	**% (95% CI)**	**Advanced neoplasia % (95% CI)**	**CRC % (95% CI)**	**Advanced neoplasia *n***	**CRC *n***	**Advanced neoplasia % (95% CI)**	**CRC % (95% CI)**	**Advanced neoplasia %**		**CRC %**		**Advanced neoplasia *n***	**CRC *n***
										***n***	**(95% CI)**	***n***	**(95% CI)**		
gFOBT		65	2.8 (2.2–3.6)	45 (33–58)	10 (4–20)	2.2	10.3	98.5 (97.9–99.0)	97.6 (94.8–98.9)	28	1.2 (0.8–1.7)	6	0.3 (0.1–0.6)	84	392
FIT	50	241	8.1 (7.2–9.1)^*^	42 (36–49)	7 (4–11)	2.4	14.1	95.5 (94.5–96.3)^*^	92.9 (88.8–95.5)^*^	95	3.2 (2.6–3.9)^*^	16	0.5 (0.3–0.9)	31^*^	186
	75	170	5.7 (4.9–6.6)^*^	49 (42–57)	9 (5–14)	2.0	11.6	97.2 (96.5–97.7)^*^	95.0 (91.8–97.0)^*^	80	2.7 (2.2–3.3)^*^	14	0.5 (0.3–0.9)	37^*^	213
	100	143	4.8 (4.1–5.6)^*^	53 (45–61)	10 (6–17)	1.9	9.8	97.8 (97.2–98.2)^*^	95.8 (93.2–97.5)	73	2.5 (2.0–3.1)^*^	14	0.5 (0.3–0.8)	41^*^	213
	125	128	4.1 (3.4–4.9)^*^	57 (48–65)	11 (6–17)	1.8	9.5	98.2 (97.7–98.6)	96.3 (93.8–97.8)	70	2.3 (1.9–3.0)^*^	13	0.4 (0.3–0.8)	43^*^	229
	150	120	4.0 (3.4–4.8)^*^	60 (51–69)	11 (7–19)	1.7	8.8	98.4 (98.0–98.7)	96.6 (94.2–98.0)	69	2.3 (2.8–2.9)^*^	13	0.4 (0.3–0.8)	43^*^	229
	175	107	3.6 (3.0–4.3)^*^	63 (53–72)^*^	12 (7–20)	1.6^*^	8.5	98.7 (98.3–99.0)	97.0 (95.0–98.3)	64	2.2 (1.7–2.7)^*^	12	0.4 (0.3–0.8)	46^*^	248
	200	103	3.5 (2.9–4.2)^*^	62 (52–71)^*^	12 (7–20)	1.6^*^	8.2	98.8 (98.4–99.0)	97.1 (95.0–98.4)	61	2.1 (1.6–2.6)^*^	12	0.4 (0.3–0.8)	49^*^	248

CRC=colorectal cancer; FIT=immunochemical faecal occult blood test; gFOBT=guaiac-based faecal occult blood test; NNscope=number needed to scope to detect one screenee with an advanced neoplasia; NNscreen=number needed to screen to detect one screenee with an advanced neoplasia; PPV=positive predictive value; TC=total colonoscopy.

^*^P<0.05 compared with gFOBT; advanced neoplasia: adenoma ⩾10 mm, villous component (⩾25% villous) or high-grade dysplasia; CRC.

**Table 2:** Colonoscopic findings per screenee according to the haemoglobin levels of the positive FIT

	**Haemoglobin level in ng ml^−1^**			
	**50–100 *n* (%)**	**100–150 *n* (%)**	**150–200 *n* (%)**	**⩾200 *n* (%)**
Total screenees	89 (100)	22 (100)	17 (100)	98 (100)
No findings	37 (42)	11 (50)	4 (23)	19 (19)
Non-neoplastic polyp	8 (9)	1 (5)	3 (18)	3 (3)
Non-advanced adenomas	22 (25)	6 (27)	2 (12)	15 (15)
Advanced adenomas	20 (22)	3 (14)	7 (41)	49 (49)
CRC	2 (2)	1 (5)	1 (6)	12 (12)
Advanced neoplasia	22 (25)	4 (18)	8 (47)	61 (61)

CRC=colorectal cancer; FIT=immunochemical faecal occult blood test.

Advanced adenoma: adenoma ⩾10 mm, villous component (⩾25% villous) or high-grade dysplasia; CRC.
